# Estimating Compressive and Shear Forces at L5-S1: Exploring the Effects of Load Weight, Asymmetry, and Height Using Optical and Inertial Motion Capture Systems

**DOI:** 10.3390/s24061941

**Published:** 2024-03-18

**Authors:** Iván Nail-Ulloa, Michael Zabala, Richard Sesek, Howard Chen, Mark C. Schall, Sean Gallagher

**Affiliations:** 1 Department of Industrial and Systems Engineering, Auburn University, Auburn, AL 36849, USA; ivan.nail@auburn.edu (I.N.-U.); sesek@auburn.edu (R.S.); szg0036@auburn.edu (S.G.); 2 Institute of Industry and Management, Universidad Austral de Chile, Puerto Montt 5480000, Chile; 3 Department of Mechanical Engineering, Auburn University, Auburn, AL 36849, USA; zabalme@auburn.edu; 4 Department of Industrial and Systems Engineering and Engineering Management, The University of Alabama at Huntsville, Huntsville, AL 35899, USA

**Keywords:** motion capture, lifting biomechanics, ergonomics, wearables, Xsens, musculoskeletal modeling

## Abstract

This study assesses the agreement of compressive and shear force estimates at the L5-S1 joint using inertial motion capture (IMC) within a musculoskeletal simulation model during manual lifting tasks, compared against a top-down optical motion capture (OMC)-based model. Thirty-six participants completed lifting and lowering tasks while wearing a modified Plug-in Gait marker set for the OMC and a full-body IMC set-up consisting of 17 sensors. The study focused on tasks with variable load weights, lifting heights, and trunk rotation angles. It was found that the IMC system consistently underestimated the compressive forces by an average of 34% (975.16 N) and the shear forces by 30% (291.77 N) compared with the OMC system. A critical observation was the discrepancy in joint angle measurements, particularly in trunk flexion, where the IMC-based model underestimated the angles by 10.92–11.19 degrees on average, with the extremes reaching up to 28 degrees. This underestimation was more pronounced in tasks involving greater flexion, notably impacting the force estimates. Additionally, this study highlights significant differences in the distance from the spine to the box during these tasks. On average, the IMC system showed an 8 cm shorter distance on the *X* axis and a 12–13 cm shorter distance on the *Z* axis during lifting and lowering, respectively, indicating a consistent underestimation of the segment length compared with the OMC system. These discrepancies in the joint angles and distances suggest potential limitations of the IMC system’s sensor placement and model scaling. The load weight emerged as the most significant factor affecting force estimates, particularly at lower lifting heights, which involved more pronounced flexion movements. This study concludes that while the IMC system offers utility in ergonomic assessments, sensor placement and anthropometric modeling accuracy enhancements are imperative for more reliable force and kinematic estimations in occupational settings.

## 1. Introduction

Lower back disorders are a significant health problem affecting one out of four workers in the United States [1]. Manual material handling tasks, such as lifting, impose high compression and shear forces on the spine [2]. Spinal compression and shear forces can cause damage to spinal segments and the surrounding tissues [3,4]. Understanding lower back loading is a central area of study in ergonomics, such as estimating moments and forces (compressive and shear) experienced by the lumbar spine during physical work. In order to protect workers and establish ergonomic safety limits for job design and intervention, the National Institute for Occupational Safety and Health (NIOSH) determined the compressive force limit for the lumbar vertebrae to be 3.4 kN [5]. Also, there have been studies on the tolerance of the lumbar spine to shear force, contributing to a recommended limit of 1 kN [6,7,8]. However, assessing spinal loading in vivo is challenging and rarely performed due to the method’s invasiveness [2]. Furthermore, the assessment is typically confined to controlled laboratory environments, limiting the ability to evaluate spinal loading in real-world field settings such as live production environments [9].

Musculoskeletal simulations and dynamic modeling programs that can predict forces and moments acting on the musculoskeletal system during movement are plausible options in biomechanics [10,11]. These simulations typically involve the creation of a computer-based model of the human body, including its bones, joints, ligaments, and muscles. Using equations of motion, principles of mechanics, and optimization methods, the model can predict the forces and moments acting on the musculoskeletal system during movement, providing insights into the loading experienced by different body tissues [12].

Using optical motion capture (OMC) technology, researchers can effectively capture kinematic data, such as the segment orientation, position, linear and rotational dynamics, and joint angles, by adhering to International Society of Biomechanics guidelines, which involve placing reflective markers on specific anatomical sites [13,14]. Additionally, force plates (FPs) allow kinetic analysis by recording the ground reaction forces (GRFs) and their associated moments. Yet, the real-world application of these tools in professional settings often faces complexities or infeasibility. This prompts the need to consider alternative technologies more attuned to the dynamic demands of work environments [15].

Additionally, to address the “portability” limitation of optical motion capture systems and laboratory-based equipment, some wearable measurement systems have been developed for ambulatory assessment of backloading [16,17]. However, they can be bulky and impractical for highly dynamic conditions. In order to measure the motion associated with physical work in dynamic workplaces, inertial motion capture (IMC) usage has increased during the last decade [15,18].

Kinematic data obtained from IMC systems have been validated in several studies in laboratories against data obtained from OMC systems [19,20,21,22,23,24]. Estimating kinetic variables such as backloading from IMC systems has also been validated in laboratories by using force platforms or instrumented shoes to measure the ground reaction forces [20,25,26,27,28]. Recent advancements in IMC technology, such as consistency of performance over time, accuracy, and cost efficiency [21,29], and the availability of methods to predict ground reaction forces and moments from the segment kinematics and dynamical properties have provided new opportunities for field assessment [20,30,31,32,33]. Larsen et al. [20] observed moderate-to-excellent correlations for the intraclass correlation coefficients for compression and anteroposterior shear forces at the L4–L5 joint, ranging from 0.65 to 0.92 during symmetrical and asymmetrical lifting. For the analyzed trials, however, the IMC-based model underestimated the trunk flexion compared with those measured with the OMC system, with a reported RMSE ranging from 12.43 to 16.54 degrees. The combination of ambulatory IMC measurements and musculoskeletal modeling for estimating spinal loads during manual material handling needs further evaluation before it can be used in the field. In particular, no studies have systematically evaluated the effects of key ergonomic variables such as the load mass [34,35,36,37], lifting and lowering height [38,39], asymmetry [8,40], or their potential interactions.

The current study aims to critically investigate the capability of an inertial motion capture (IMC) system in accurately assessing compression and anteroposterior shear forces at the L5-S1 joint during lifting and lowering tasks. The tasks are varied across three primary factors: the mass of the load, the degree of asymmetry, and the height at which the load is handled. Within this context, the study is guided by two distinct objectives:

1. Assess the agreement between the IMC- and OMC-based models in estimating lumbar spine kinetics and kinematics.
2. Systematically evaluate the influence of the load mass, asymmetry degree, and lifting height on the kinematics and kinetics within and between both IMC and OMC systems.

Clarifying and examining these objectives are an essential part of evaluating the effectiveness of IMC systems and understanding the factors that may impact their estimates when assessing biomechanical loads during manual handling tasks. Additionally, trunk rotation, flexion, and lateral bending were compared to better understand the differences in the force estimates. A measure of the distance between the handled box and the L5-S1 joint’s center was compared between systems as a comprehensive estimation of the segment length differences.

## 2. Materials and Methods

A gender-balanced sample of 36 participants (19–55 years old; mean height = 173.54 ± 7.5 cm; mean body mass = 72.78 ± 12.1 kg) was recruited. The participants were from the Auburn University student body and the local Auburn-Opelika community. The inclusion criteria included (1) no history of physician-diagnosed MSD, injury, or surgery in the lower back, (2) absence of lower back pain during the previous six months, and (3) no history of a physician-diagnosed neurodegenerative disorder that may affect movement (e.g., Parkinson’s disease or multiple sclerosis). The study was approved by the Institutional Review Board of Auburn University (Protocol No. 16-211 MR 1606).

### 2.1. Participant Preparation and Data Collection

**Figure 1:**
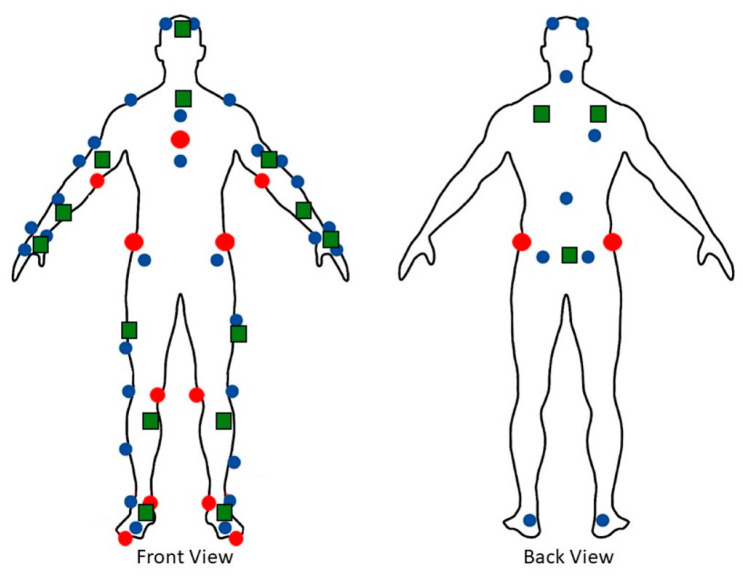
Full-body participant set-up, adapted Plug-in Gait marker set, and IMU sensors, with regular markers (blue circles), additional markers (red circles), and IMU sensors (green squares).

**Figure 2:**
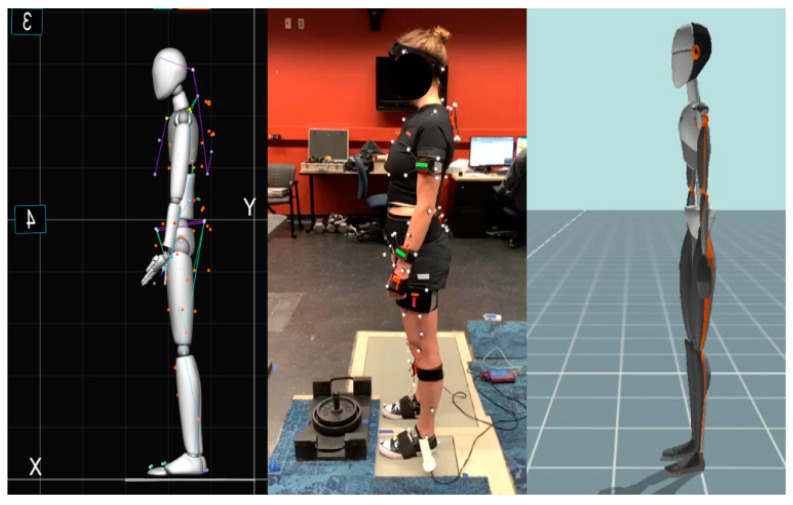
(**left**) OMC model avatar. (**middle**) Participant. (**right**) IMC model avatar.

Several anthropometric measurements were obtained, including the heights, weights, and lengths and circumferences of several major body links following established guidelines [41]. The participants were fitted with 17 IMU Xsens Awinda sensors (Xsens Technologies, Enschede, The Netherlands). Each IMU is a small, wireless, battery-powered unit that measures and stores the acceleration, angular velocity, and magnetic field information. The devices were secured using a combination of elastic neoprene straps or hypoallergenic athletic tapes. In addition to the IMUs, the participants wore small, reflective motion capture markers used as a “gold-standard” reference for body segment positions. The reflective markers’ trajectories were recorded with 16 cameras (Optitrack Prime 13, Natural Point, Inc., Corvallis, OR, USA). For this experiment, a total of 50 reflective markers were used. A full-body Plug-in Gait marker set was used to define the reflective markers’ locations; 11 markers were added to the 39 marker set, including on the insides of the knees, elbows, and ankles, tops of the feet, and left and right sides of the pelvis (Figure 1). The anthropometric data were used to build a rigid link biomechanical model using the information collected from the sensors. The model was compared against the one collected from the reflective markers. Major anthropometric measurements are summarized in Table 1. Figure 2 illustrates the experimental settings and the avatars from the respective systems (Video S1).

### 2.2. System Calibration

Standard procedures were used to calibrate the OMC and IMC systems. Segment calibration of the IMC system was necessary to align the motion trackers to the participants’ segments. The procedure consisted of the participant holding a neutral posture for a few seconds and then walking straight forward and back to the point where they started. The OMC system was calibrated before each data collection session, defining the capture volume and the global coordinate system’s orientation and origin.

### 2.3. Musculoskeletal Model

The biomechanical assessment was performed using the musculoskeletal modeling software Anybody™ V.7.3.1 (AnyBody Technology, Aalborg, Denmark). The OMC-based template used for the analysis was the Plug-in-Gait-MultiTrial-Standing set. For the IMC system, the template used was BVH_Xsens. The models were scaled according to the manually measured segment dimensions, which were inputted into the IMC software (v 2019.2) before initiating the data collection sessions. The BVH files from the IMC software were exported, containing information on the kinematic model static pose and the absolute position and orientation of the root pelvis segment as well as the joint angles between the segments for each time frame [42]. The Anybody Model Repository (AMR) used in this study to process BVH files was AMR v2.3.1. The compressive (proximo-distal (PD)) and shear (antero-posterior (AP)) forces were considered for the analysis. Medio-lateral (ML) shear forces were not considered due to their difference and smaller magnitude compared with the other two forces [43]. The load lifted (box) was modeled in the software using the distance between the hands and the dimensions of the box as references, with the dimensions of the actual box being lifted being 10 cm height, 25 cm width, 40 cm length. 

### 2.4. Data Synchronization and Processing

Motion analysis was performed by using the OMC and IMC systems simultaneously. OMC data were sampled at 120 Hz, while IMC data were sampled at 60 Hz. The forces and kinematics were bidirectionally low-pass filtered with a second-order Butterworth filter at 7 Hz for the forces system and 3 Hz for the kinematics. Gaps in the OMC markers’ trajectories were filled using cubic interpolation methods in the Motive™ software v2.1.1. The force estimates for the OMC-based model were obtained using a top-down approach. In contrast, the IMC-based estimates were obtained through kinematics using the model utilized by Karatsidis et al. [42].

### 2.5. Experimental Procedure

The 36 participants were instructed to perform different manual material handling tasks in a laboratory environment, where kinematic and kinetic data were collected. The duration of the trials varied from 10 to 30 s, depending on the participant. Each participant performed three trials with a 2 min rest period in between. The participants were instructed to lift boxes (similar to a standard milk crate) with handles consisting of three loads—10 lbs (4.54 kg), 20 lbs (9.07 kg), and 30 lbs (13.61 kg)—both symmetrically and asymmetrically (i.e., 0°, 30°, and 60° degrees from the sagittal plane to the left) to three different shelf heights (60 cm, 100 cm, and 140 cm). Each participant completed three different combinations using the lifting technique of their preference. The tested combinations were labeled using the following terminology: LXX (XX = load of 10, 20, or 30 lbs), AYY (YY = angle of 0-, 30, or 60 degrees asymmetry), and HZZ (ZZ = height of 60, 100, or 140 cm). For example, L20_A30_H140 would represent a 20 pound load picked up from a sagittal angle of 30° and lifted to 140 cm. The loads and heights were randomized within the levels of angle for 27 combinations (every combination was performed 4 times, meaning 108 trials).

### 2.6. Measurements

**Figure 3:**
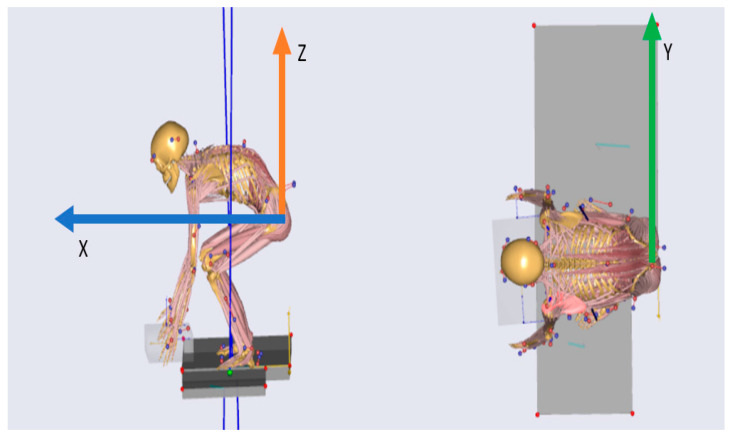
Distance of box to spine illustration from Plug-in Gait model template in Anybody™.

The L5-S1 compression and shear forces, flexion, lateral bending, and rotation angles and the distance between the joint and the box on the X, Y, and Z axes were determined. These variables were measured in units of Newtons (N), degrees (°), and meters (m), respectively. The distance from the center of the L5-S1 joint was measured as indicated in Figure 3. The figure displays an example with the following coordinates: (X_1_, Z_1_, Y_1_) = (0.73, −0.31, −0.01), with X_1_ = 0.73 m for the box in front of the sacrum, Z_1_ = 0.31 m for the box under the sacrum, and Y_1_ = 0.01 m for the box to the left of the sacrum.

#### Data Analysis

Boxplots, kernel density estimate (KDE) plots, simple linear regression analyses, and Bland–Altman plots were used to compare the level of agreement between the systems. A 3^3^ randomized block partially confounded design (RBPF) with blocks of a size of three was used to compare the peak forces and angles, as well as the root mean square errors (RMSEs) of the time series of the forces and angles between the systems. Confounded factorial designs are particularly appropriate if an interaction is expected to be negligible. The interactions can be confounded with groups, reducing the block size without sacrificing power in evaluating the treatments [44]. Due to the nature of the experimental design technique, the groups (four groups of nine participants each) and the main effects (load, angle, height, and their possible interactions) were analyzed to find potentially significant differences. A type I error rate of 0.05 was used for all tests. Pairwise comparisons were performed using Tukey’s test to analyze the RMSE and range for the main effects and interactions found to be significant. The effect sizes were calculated to measure the strength of the relationship between variables. Omega-squared (ω^2^) is recommended for complex designs with multiple variables [45]. Statistical tests were conducted using Python (v 3.10.11).

## 3. Results

**Figure 4:**
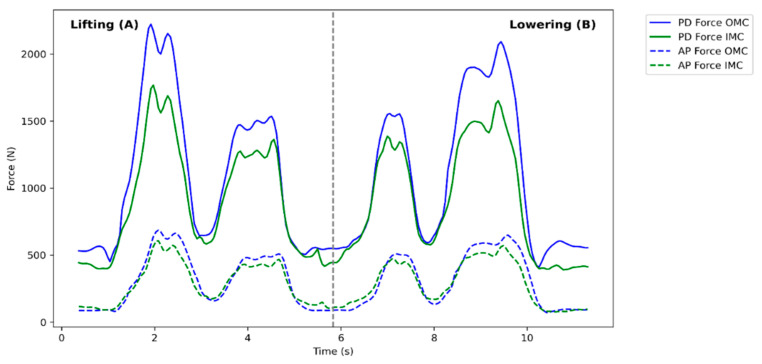
Compressive (PD) and shear (AP) forces during one of the lifting (**A**) and lowering (**B**) trials.

The results were divided into two main categories, with the first showing the agreement between systems, presenting descriptive statistics of the compressive and anteroposterior shear forces (Table A1, Appendix A), joint angles (Table A2), and peak distances from the box to the spine (Table 2) and the second showing the statistical effect of the analyzed variables, the results of the experimental design (RBPF), and post hoc analyses. Figure 4 shows an example of a full-duration trial considering the force estimates for lifting and lowering. The example corresponds to a female participant lifting a load of 10 lbs (L10) with no asymmetry angle (A00) and a height of 1 m (H100). In both the lifting and lowering diagrams, two distinct peaks are evident. The more prominent peak typically corresponds to lifting or setting down the load from or onto the floor. Conversely, the secondary peak is commonly associated with placing or retrieving the load onto or from the shelf.

### 3.1. Forces

#### 3.1.1. Compressive Forces

**Figure 5:**
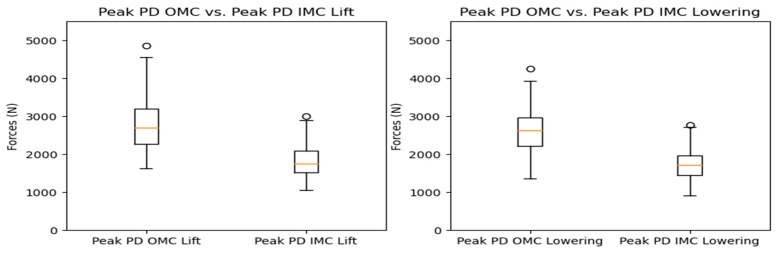
Box plots for peak compressive force for the OMC and IMC systems for the lifting (**left**) and lowering (**right**) tasks. Circles represent outliers.

**Figure 6:**
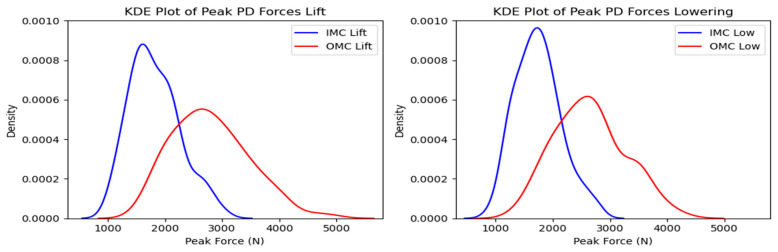
KDE plots for peak compressive force for the OMC and IMC systems for the lifting (**left**) and lowering (**right**) tasks.

**Figure 7:**
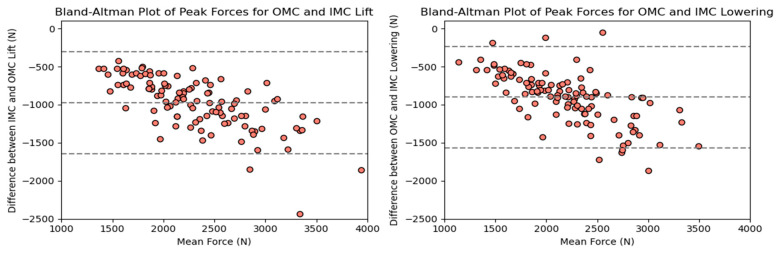
Bland–Altman plots for OMC and IMC system peak compressive force estimates for the lifting (**left**) and lowering (**right**) tasks.

The compressive force estimates varied significantly between systems. The OMC results were, on average, 34% higher than the IMC estimates. Figure 5 shows the resultant box plots with the force estimates, where the difference increased as the observed forces increased. The KDE plots display the distribution of observations, shown in Figure 6. Due to the comparison of distribution shapes, it is noticeable that the OMC estimates showed a wider range of values than the IMC estimates for both the lifting and lowering scenarios. Figure 7 presents the Bland–Altman plots, highlighting a trend wherein increases in the magnitude of the force estimates increased the differences in agreement between the systems.

**Figure 8:**
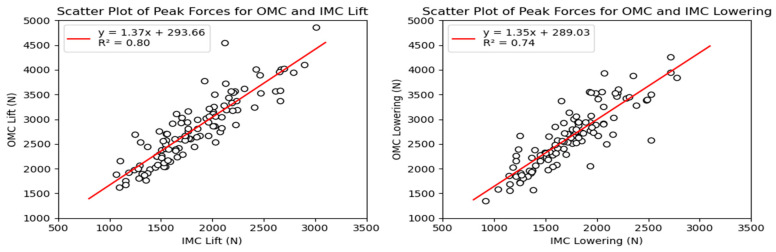
Scatter plots for OMC and IMC systems’ peak compressive force estimates for the lifting (**left**) and lowering (**right**) tasks.

The simple linear regression analysis for the tasks is shown in Figure 8. For the lifting and lowering tasks, the regression line equations suggest a multiplication of 1.37 (95% CI: 1.24–1.51) and 1.35 (95% CI: 1.20–1.51) for the force estimates, with 293.66 (95% CI: 293.52–293.79), and 289.03 N (95% CI: 288.88–289.18) added as the intercepts, respectively.

**Figure 9:**
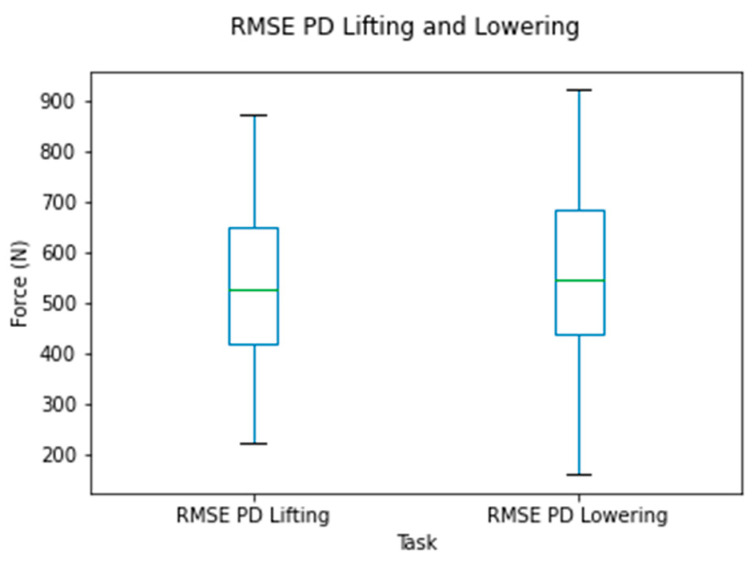
Box plots for the RMSE of the compressive force for the OMC and IMC systems for the lifting and lowering tasks.

The RMSE of the compressive forces for the time series data for all observations is shown in Figure 9. The RMSE varied mainly between 420 and 756 N (from the 25th to 90th percentiles) for the lifting and lowering tasks.

**Figure 10:**
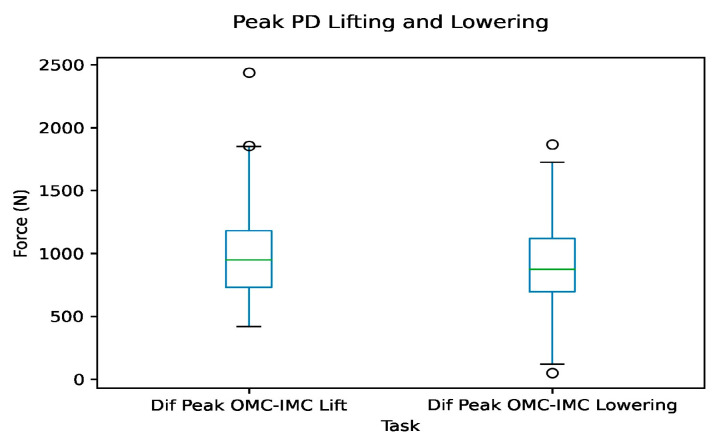
Box plots of peak compressive force differences between systems for the lifting and lowering tasks. Circles represent outliers.

The peak compressive force differences were also calculated, mostly falling between 696 and 1397 N, with most observations being between the 25th and 90th percentiles, as seen in Figure 10.

#### 3.1.2. Shear Forces

**Figure 11:**
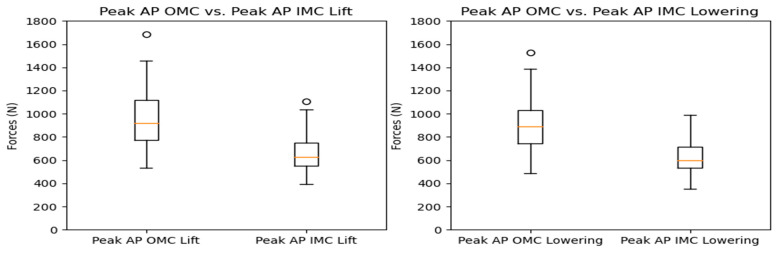
Box plots for peak shear force for the OMC and IMC systems for the lifting (**left**) and lowering tasks (**right**). Circles represent outliers.

**Figure 12:**
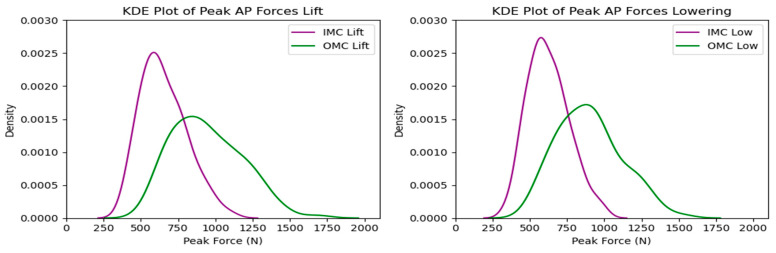
KDE plots for peak shear force for the OMC and IMC systems for the lifting (**left**) and lowering (**right**) tasks.

**Figure 13:**
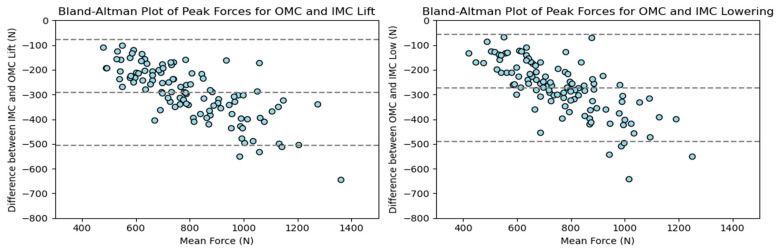
Bland–Altman plots for OMC and IMC system peak shear force estimates for the lifting (**left**) and lowering (**right**) tasks.

As with the compressive forces, the shear forces exhibited significant variation between systems, with an average difference of 30% for the lifting and lowering tasks. The maximum peak shear force observed was around 1600 N in the OMC system, while the IMC system had a maximum peak shear force of 1200 N for both the lifting and lowering tasks. Differences between 200 and 400 N were observed for the values between the 25th and 90th percentiles. Figure 11 displays the shear force estimates for each system, with noticeable differences between the estimates, especially at the higher end of the observed forces. Additionally, the probability density functions for the observations displayed in the KDE plots (Figure 12) showed a similar situation to the one observed for the compressive forces, with the OMC estimates having a wider range of values and generally higher estimates than the IMC-based estimates. As for the compressive force estimates, the Bland–Altman plots in Figure 13 reveal noticeable differences between the observations for the shear forces, highlighting the trend of higher differences with higher force estimates.

**Figure 14:**
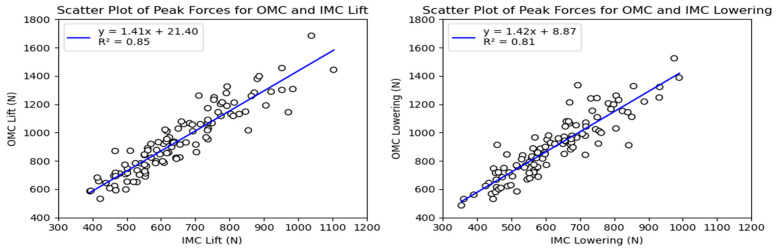
Scatter plots for OMC and IMC system peak shear force estimates for the lifting (**left**) and lowering (**right**) tasks.

The simple linear regression analysis for the tasks is shown in Figure 14. For the lifting and lowering tasks, the regression line equations suggested slopes of 1.41 (95% CI: 1.30–1.53) and 1.42 (95% CI: 1.29–1.56) with intercepts of 21.40 (95% CI: 21.29–21.52) and 8.87 N (95% CI: 8.74–9.00), respectively.

**Figure 15:**
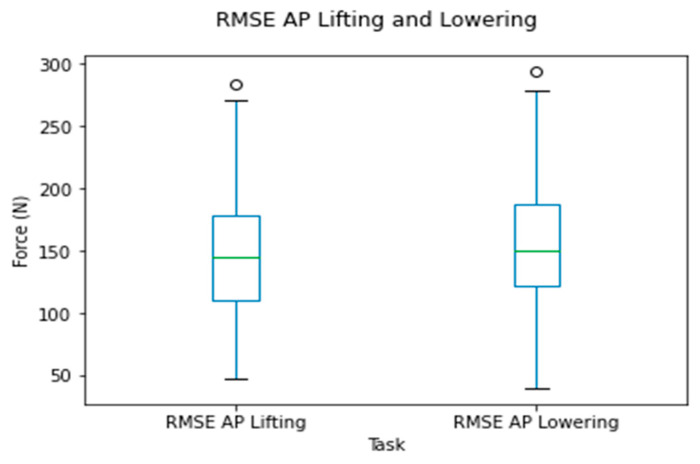
Box plots for the RMSE of the shear forces between systems for the lifting and lowering tasks. Circles represent outliers.

The RMSE of the shear forces for the time series data for all observations is shown in Figure 15. The RMSE varied mainly between 109 and 225 N (25th to 90th percentiles) for the lifting and lowering tasks, respectively.

The peak shear force differences were also calculated, mostly falling between 205 and 435 N, with most observations being between the 25th and 90th percentile.

### 3.2. Joint Angles and Distance from the Spine to the Load

#### 3.2.1. Flexion

**Figure 16:**
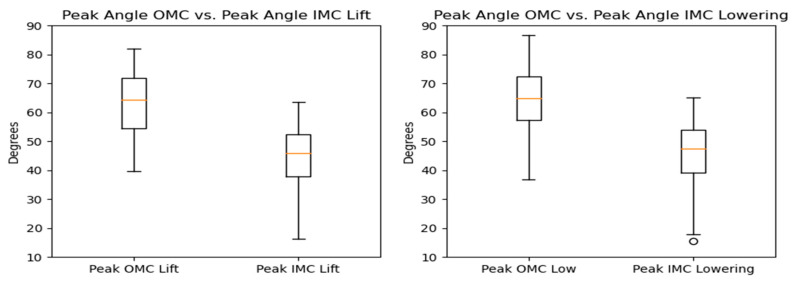
Box plots for the peak flexion angle for the OMC and IMC systems for the lifting (**left**) and lowering (**right**) tasks. Circles represent outliers.

**Figure 17:**
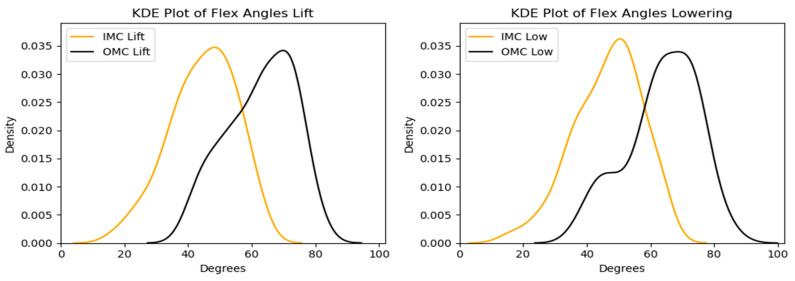
KDE plots for the peak flexion angle for the OMC and IMC systems for the lifting (**left**) and lowering (**right**) tasks.

**Figure 18:**
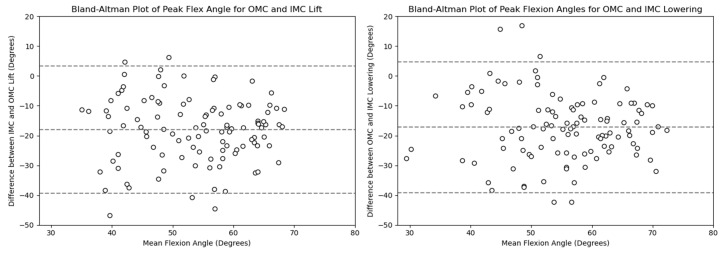
Bland–Altman plots for the OMC and IMC system peak flexion angle estimates for the lifting (**left**) and lowering (**right**) tasks.

The peak flexion angle comparison for every system showed that the OMC-based estimates were higher than the IMC-based ones by around 14–18 degrees. Figure 16 and Figure 17 display the differences observed. Figure 18 shows the Bland–Altman plots, where it is displayed that the mean differences were 17.9 and 17.1 degrees for the lifting and the lowering tasks, respectively.

The RMSE ranged from 2.45 to 28.54 degrees for both the lifting and lowering tasks, with most values falling between the 25th and 75th percentiles (7.06–13.13 degrees).

#### 3.2.2. Lateral Bending

**Figure 19:**
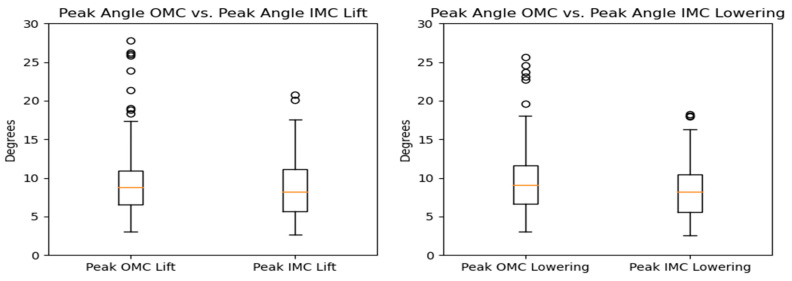
Box plots for the peak lateral bending angle for the OMC and IMC systems for the lifting (**left**) and lowering tasks (**right**). Circles represent outliers.

**Figure 20:**
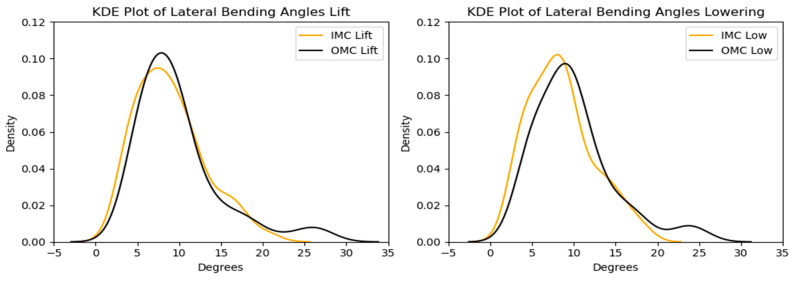
KDE plots for the peak lateral bending angle for the OMC and IMC systems for the lifting (**left**) and lowering (**right**) tasks.

**Figure 21:**
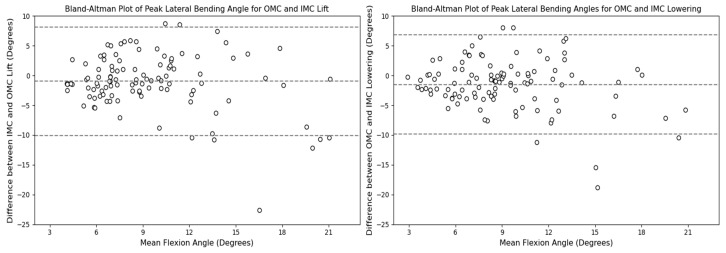
Bland–Altman plots for the OMC and IMC system peak lateral bending angle estimates for the lifting (**left**) and lowering (**right**) tasks.

The comparison of peak lateral bending angles across the different systems revealed that the estimates based on the OMC and IMC methods were generally similar, with median values of approximately 8.5 degrees falling within the interquartile range for all scenarios. However, the OMC-based estimates were higher than those of the IMC system for the lifting and lowering tasks. The observed differences are illustrated in Figure 19 and Figure 20. Bland–Altman plots were generated, and they are displayed in Figure 21. The plots indicate that the mean differences in the peak lateral bending angles between the two systems were approximately −1 degree for the lifting and lowering tasks. In the lifting and lowering tasks, the RMSE ranged from 0.92 to 11.42 degrees, with values between the 25th and 75th percentiles ranging from 3.79 to 5.16 degrees.

#### 3.2.3. Rotation

**Figure 22:**
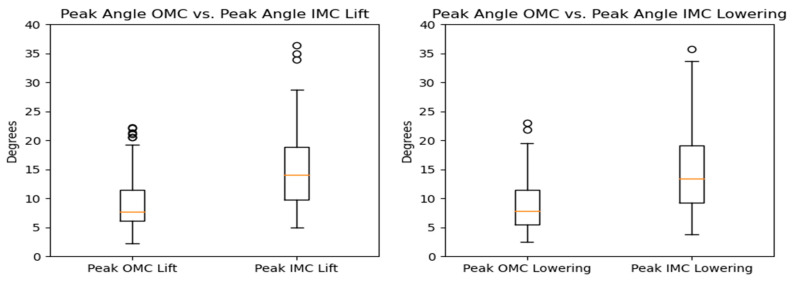
Box plots of the peak rotation angle for the OMC and IMC systems for the lifting and lowering tasks. Circles represent outliers.

**Figure 23:**
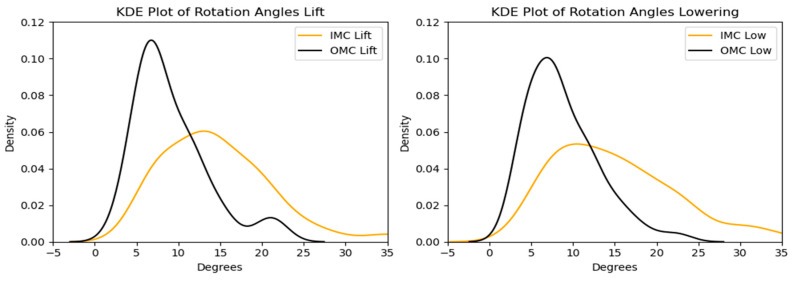
KDE plots for the peak rotation angle for the OMC and IMC systems for the lifting (**left**) and lowering (**right**) tasks.

**Figure 24:**
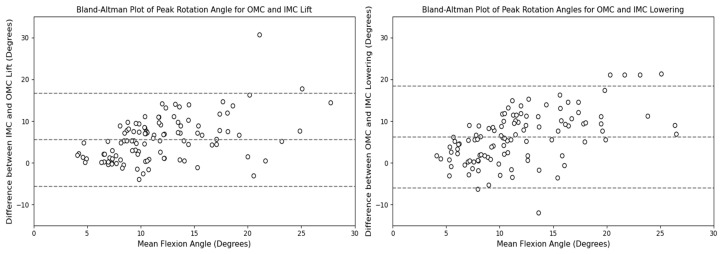
Bland–Altman plots for the OMC and IMC system peak rotation angle estimates for the lifting (**left**) and lowering (**right**) tasks.

In terms of the peak rotation angles, the comparison between the OMC and IMC methods showed that the IMC estimates were generally higher for the lifting and lowering tasks, with median values of approximately 7.74 and 13.44 degrees, respectively. These differences are illustrated in Figure 22 and Figure 23. Bland–Altman plots were generated, as shown in Figure 24, to assess the agreement between the two systems. The plots reveal that the mean differences in the peak rotation angles between the OMC and IMC systems were 5.51 degrees (limits of agreement: −5.65, 16.67) for the lifting task and 6.19 degrees (limits of agreement: −5.98, 18.35) for the lowering task. The lifting and lowering tasks had RMSE values ranging from 0.94 to 16.31 degrees, with the 25th to 75th percentile values ranging from 3.61 to 7.77 degrees.

#### 3.2.4. Distance from the Spine to the Load

**Figure 25:**
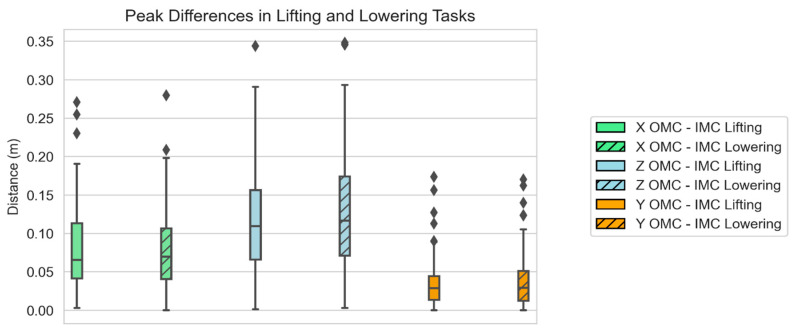
Box plots of the peak distance differences at peak force for the OMC and IMC systems for the lifting and lowering tasks. Diamonds represent outliers.

The distance between the spine and the load was analyzed on the *X*, *Y*, and *Z* axes. The results are presented in Figure 25. The mean difference in the peak distance was 7.90 cm for the lifting task and 7.93 cm for the lowering task on the *X* axis, with a range from 0 to 27.94 cm. The mean difference in the peak distance on the *Z* axis was higher, being 11.51 cm for the lifting task and 12.53 cm for the lowering task, and the range was 0.1–34.80 cm. The *Y* axis showed smaller differences, with a mean of 3.41 cm for the lifting task, 3.71 cm for the lowering task, and a range of 0–17.34 cm.

### 3.3. Statistical Analysis

The RBPF 3^3^ ANOVA results were divided into forces and joint angles. The analyses compared the peaks (forces and angles), RMSEs between systems for the time series, and peak differences for the lifting and lowering tasks. The summary of the forces for the analysis of the effect of a load’s weight, height, and asymmetry and their potential interactions are displayed in Table 3 and Table 4.

#### 3.3.1. Compressive Forces

The statistical analysis revealed significant differences between the participant groups across all scenarios. Furthermore, among the three main factors investigated, the load was significant for the individual peaks, the RMSE, and the peak differences between the systems in both the lifting and lowering tasks. Post hoc tests using Tukey’s method demonstrated significant differences between all load levels, with the greatest difference observed between L10 and L30. Additionally, height was found to be a significant factor for estimates of the RMSE in both tasks, with H60 showing significant differences from H100 and H140. The observed trends were consistent across both the lifting and lowering tasks.

#### 3.3.2. Shear Forces

The statistical analysis revealed significant differences between the participant groups across all scenarios, as presented in Table 3 and Table 4. Furthermore, among the three main factors investigated, the load was significant for the individual peaks, the RMSE, and the peak differences between systems in both the lifting and lowering tasks. Post hoc tests using Tukey’s method demonstrated significant differences between all load levels, with the greatest difference observed between L10 and L30. Additionally, height was found to be a significant factor for the estimates of the RMSE in both tasks, with H60 showing significant differences from H100 and H140. The observed trends were consistent across both the lifting and lowering tasks.

#### 3.3.3. Joint Angles

The ANOVA results for the joint angles are summarized in Table 5 and Table 6.

##### Flexion

The flexion and extension angle analysis revealed significant differences between the groups for both systems. The lifting task also showed significant effects from the load factor on the peak IMC (*p* = 0.028) and the angle factor on the peak OMC (*p* = 0.003) and IMC (*p* = 0.014), with increasing peak flexion angle values as the level of the angle factor increased and A00 being significantly different from A60. However, the angle did not affect any of the peaks for the lowering task, but the height did for the peak IMC (*p* = 0.017), with H100 being significantly different from H140. The RMSEs for both tasks did not show significant differences. Although significant differences were not observed when evaluating the peak differences for the lifting task, a significant effect from the height was observed for the lowering task (*p* = 0.002). Tukey’s test indicated that H140 was significantly different from H060 and H100.

##### Lateral Bending

In terms of lateral bending, both the RMSEs and peak differences for the lifting and lowering tasks did not exhibit any significant effects. However, the peaks for both systems in both tasks demonstrated a significant association with the angle (*p* ≤ 0.001). Further analysis using Tukey’s test revealed significant differences between every angle level for the IMC (lifting and lowering) and OMC (lowering) set-ups. Additionally, A60 exhibited a significant difference from A00 and A30 for the OMC set-up in the lifting task. Regarding the peak OMC in the lowering task, the load also displayed significance (*p* = 0.020). Tukey’s test indicated that L10 was significantly different from L20 and L30.

##### Rotation

Regarding the rotation angle, the analysis revealed significant effects from groups on the peak IMC and peak differences for both the lifting and lowering tasks. Furthermore, the load factor was found to be significant only for the peak differences in the lowering task (*p* = 0.0049), specifically with L10 showing a significant difference from L20.

Additionally, the angle factor demonstrated significance for the peaks in both the OMC and IMC systems for both tasks (*p* ≤ 0.001), with each angle level displaying significant differences. The peak difference between systems in the lowering task also exhibited a significant association with the angle factor (*p* ≤ 0.001). Further analysis using Tukey’s test revealed that A60 significantly differed from A00 and A30, indicating higher differences as the angle level increased. Height was observed to be a significant factor in the lifting task, specifically in the estimates of the peak OMC (*p* = 0.008). Post hoc analysis using Tukey’s test revealed that H140 significantly differed from H100 and H060. Similarly, in the peak difference between systems, height was found to be a significant factor (*p* = 0.018), with post hoc analysis indicating that H140 was significantly different from H060.

## 4. Discussion

This study assessed the agreement of compressive and shear force estimates at the L5-S1 level obtained by a musculoskeletal model using data from an ambulatory measurement system consisting of inertial motion capture (IMC) during manual lifting. The outcomes of the IMC-based model were compared against a top-down optical motion capture (OMC)-based model.

### 4.1. Agreement between Force Estimates

The results revealed significant differences between the compressive (PD) and anteroposterior (AP) shear force estimates obtained from the OMC and IMC systems. On average, the IMC estimates were 34% (975.16 N) lower for the compressive forces and 30% (291.77 N) lower for the shear forces. Additionally, the values obtained from the OMC system exhibited a broader range than those from the IMC system for both force estimates. These discrepancies between the IMC and OMC systems carry practical implications, particularly when the estimates are utilized to contextualize activities within ergonomic thresholds, such as the recommended limits for compression and shear forces during manual material handling tasks, which are 3400 N and 1000 N, respectively [5,6,8,35]. The linear regression analysis of the force estimates (Figure 8 and Figure 14) indicates that obtaining a PD force of 2300 N with the IMC system would be equivalent to approximately 3400 N. In comparison, an AP force of 700 N would be equivalent to approximately 1000 N. In other words, the IMC-based forces were approximately 30% lower than the OMC estimates. Another observed trend revealed by the Bland–Altman plots (Figure 7 and Figure 13) showed that as the force estimates increased, the discrepancies between the estimates increased. Consequently, the IMC system underestimating the forces at L5–S1 could be problematic and lead to a misinterpretation of injury risk assessment in occupational settings.

In a laboratory study by Marras and Davis [8], participants were instructed to lift a 13.7 kg load (30 lbs) at various asymmetric angles using one or two hands. The compressive and shear forces at L5-S1 were measured using electromyography (EMG) and a back electrogoniometer that measured the joint angles relative to the thorax and pelvis. The results demonstrated that the compressive forces ranged from 3700 N for a symmetrical lift to 4200 N for a 60 degree angle. In contrast, our analysis of the average compression forces for the 13.7 kg load, considering different combinations of heights and angles, yielded an average of approximately 3200 N for the OMC system and 2000 N for the IMC system.

In a recent study by Skals et al. [33], musculoskeletal modeling and an IMC system were utilized to estimate the compressive and anteroposterior shear forces in manual material handling activities in the supermarket sector. The study involved handling loads ranging from 5.3 kg to 20.2 kg, considering different starting positions and shelf heights. The authors reported noteworthy force estimates for loads of 10.2 kg (reaching up to 3442 N for compression), 17.3 kg (3854 N for compression and 1113 N for shear), and 20.2 kg (4188 N for compression and 1191 N for shear). Although their results were not directly compared against an OMC model, and the loads and lifting parameters differed from those in our study, it is important to note that the observations from their IMC-based model exhibited higher magnitudes than those observed in the present study for similar loads. We suspect that these significant force discrepancies may be attributed to kinematic variations, differences in the flexion, extension, and rotation joint angles, and disparities in model scaling or segment length.

The IMC-based model template used in the current study has been reported to underestimate trunk flexion angles in previous research [20]. Larsen et al. [20] reported root mean square errors (RMSEs) between the OMC- and IMC-based models ranging from 12.43 to 16.54 degrees for symmetrical, asymmetrical, and load-transferring lifting tasks. These results align with our observations. The mean RMSE for lifting in our study was 10.92 degrees. For lowering, it was 11.19 degrees. However, it is important to note that the degree of underestimation varied with the RMSE values, being as low as 3.73 degrees for lifting and 2.45 degrees for lowering but they also reached as high as 28 degrees for both tasks.

In addition to the trunk flexion angle, Larsen et al. [20] also investigated a comparison of the lateral bending and rotation angles. They reported RMSE values ranging from 1.8 to 2.79 degrees for the lateral bending angle and 4.58 to 7.75 degrees for the rotation angle. Our study also examined these angles, and the mean values for the lateral bending angle were 4.19 degrees for lifting, 4.16 degrees for lowering, and 5.93 degrees for rotation. These findings reinforce a similar pattern of discrepancy in angle estimates, as reported in the existing literature.

To evaluate the differences in position error between the systems, the peak differences in the distance from the lifted box to L5-S1 (at peak force) were calculated. The most considerable discrepancies were observed on the *X* and *Z* axes. Specifically, there was an average difference of 8 cm on the *X* axis and 12 cm and 13 cm on the *Z* axis for lifting and lowering, respectively. Importantly, these differences indicated underestimations by the IMC system compared with the OMC system. There was no single instance where the IMC-based model’s distance exceeded that of the OMC system. These findings are consistent with a study by Faber et al. [27], which also reported similar differences between the IMC and OMC estimates in the context of biomechanical models used to estimate moments at L5-S1.

The assessment of the spine-to-box distance provided a comprehensive evaluation of the segment length, revealing a consistent underestimation, particularly in the trunk and torso, across most IMC-based models. This consistent underestimation of segment lengths, coupled with the underestimated joint angles, especially in flexion or extension, has important implications for the kinematic inputs used to estimate kinetics, potentially explaining the observed differences in forces previously presented. One potential source of this issue may be attributed to the location and placement of the sacrum sensor in the IMC system, guided by the manufacturer. Unlike the OMC system, which tracks bony landmarks to measure the trunk length, the IMC sacrum sensor is placed in a region without prominent landmarks for identification, making its placement and stability challenging. Moreover, the sacrum sensor may experience motion and upward shifts during flexion, further complicating its accurate placement and stability [20,46].

In general, the consistently shorter torso observed in the IMC-based model could be related to changes in the position of the sacrum sensor, which may be influenced by the dynamic and varied nature of the evaluated tasks that require significant forward bending. We suspect that the sacrum sensor may primarily shift upward from its original placement, impacting segment definitions. Our results indicate that the IMC-based model underestimated torso flexion, overestimated rotation, and showed similar estimates to the OMC-based system for lateral bending. These observations could be explained by the differences in the range of motion among different spinal regions. For instance, if the torso is defined above the lumbosacral joint and closer to the thoracic region, then a decrease in flexion and an increase in rotation would be expected. Researchers have observed these differences, with the flexion angle decreasing from 20 degrees at L5-S1 to 10 degrees at T11-T12 and the rotation angle increasing from approximately 5 degrees at the lumbosacral level to 9 degrees in the thoracic region [47]. Users of the IMC system should exercise caution regarding how sensor positioning affects the torso dimensions and, by extension, not just kinematics but also kinetic estimates. It may be beneficial for IMC system manufacturers to propose improved placement methods or suggest additional adjustments during pre- or post-data processing.

### 4.2. Impact of Load, Height, and Asymmetry

To our knowledge, the current study represents the first attempt to assess the differences in force and kinematics estimates between IMC and OMC systems using musculoskeletal models within a systematic statistical framework. We analyzed the results in relation to the load, height, and asymmetry levels, as well as their potential interactions. When comparing both systems, our main findings indicate that the root mean square error (RMSE) of both the compressive (PD) and anteroposterior (AP) forces were significantly influenced by the load levels and height levels. Specifically, the RMSE values were higher as the load levels increased and lower as the height levels increased (with no significant differences between H100 and H140). We attribute these higher differences at lower heights to the increased flexion angle associated with retrieving and placing the load from the shelf compared with the other heights. Since this level involved more flexion and extension movements, the differences between the OMC- and IMC-based estimates were more pronounced.

Regarding the impact of variables on the compressive and shear force estimates for each system, our findings align with previous studies [35,36,37,48,49], indicating that the load mass has the most substantial effect. However, our results differ regarding the impact of asymmetry on AP forces. While Skals et al. [49] found that load and asymmetry affected these forces, our study did not observe a significant effect of the angle on the estimates from either the IMC- or OMC-based models. A load × height interaction effect for the shear forces during the lifting tasks was observed for the IMC-based model, suggesting that higher load and height levels lead to higher AP peak forces. However, the small effect size estimate suggests that it may not be practically meaningful.

Future research should extend the comparison and analysis of kinetics and kinematics to other pertinent body parts, such as the shoulders and knees, which are known to be susceptible to MSDs [50]. Examining the forces and moments in these areas would provide a more comprehensive assessment of the overall biomechanical impact. Additionally, exploring how changes in variables such as the lifting technique, exertion speed, and postures (e.g., squatting or bent postures) can impact the forces and moments experienced by the body could provide valuable insights. Understanding these relationships would allow for more targeted and effective ergonomic interventions.

Furthermore, future studies should incorporate the assessment of muscular demands. Considering factors such as muscle activation levels and fatigue during various tasks would provide a deeper understanding of the physiological aspects of ergonomic assessment. By integrating muscular demands into the analysis, researchers can better comprehend the interaction between muscular activity, forces, and kinematics, leading to more comprehensive and accurate ergonomic evaluations, as Skals et al. [33] suggested.

In conclusion, when comparing compressive and anteroposterior shear forces using musculoskeletal modeling with OMC- and IMC-based models, significant differences were observed, making ergonomic assessment challenging. However, improving the scaling of the IMC-based model to reduce differences in the joint angle and segment length could enhance the quality of the estimates. As expected, increasing load levels significantly impacted the force estimates, while the lower lifting height showed higher force values than other levels.

### 4.3. Limitations

The present study has several limitations that should be acknowledged. Firstly, the tasks evaluated in this study focused on asymmetry levels of 0, 30, and 60 degree angles to the left of the initial box position during a two-handed lift. However, it is important to note that going beyond 60 degrees or considering right-sided asymmetry instead of left-sided asymmetry could yield different force profile trends [8]. Furthermore, the lifting technique and exertion speed were not controlled variables in the experiment, introducing potential variability in the results [22,51]. The analysis did not consider whether squatting or bent postures were utilized during load lifting, which could impact the force estimates [51].

Additionally, an acknowledged issue when using inertial measurement units (IMUs) over time is the effect of magnetic disturbance and gyroscopic drift, which can affect the reliability of the estimates [52]. Although the impact of this effect was not directly measured in our study, it is important to note that the IMC system was recalibrated between trial combinations, and the trials lasted no more than 30 s.

We utilized OMC as it is widely considered the gold standard for recording the positions of body segments in a 3D space. Reports in the literature suggest that OMC can achieve error rates below 200 μm for 97% of a 135 m^3^ capture volume [53]. However, two main things are worth mentioning. First, OMC-based estimates are prone to error from sources such as soft tissue artifacts [54]. Secondly, due to the complexity associated with the modeling and variability of the human body regarding force generation encompassing bones, muscles, tendons, and ligaments, joint torque estimates derived though inverse dynamics carry an inherent degree of uncertainty [12,55,56].

Another limitation of our study was the significant time investment required to process the data using the applied methodology. Similar observations have been reported in the recent literature [33], suggesting that musculoskeletal simulation tools may not be feasible as a standard for expedited industrial ergonomic assessments. However, as researchers and practitioners continue to benefit from the advantages of musculoskeletal modeling, advancements will likely be made to address and overcome these limitations.

## Figures and Tables

**Table 1:** Summary of participants’ anthropometry.

	Body Weight (kg)	Body Height (cm)	Shoe Length (cm)	Ankle Height (cm)	Ankle Width (cm)	Knee Height (cm)	Knee Width (cm)	Elbow Width (cm)	Shoulder Width (cm)	Shoulder Height (cm)	Hip Height (cm)	Hip Width (cm)	Arm Span (cm)
Mean	72.78	173.54	25.06	8.72	7.92	46.96	10.84	8.42	34.28	142.05	90.88	24.53	172.09
SD	12.1	7.5	1.3	10.0	6.8	9.8	6.5	10.1	3.3	6.8	8.9	2.0	9.1

**Table 2:** Summary of the differences in peak distance estimates for both motion capture systems.

Summary Measure	X OMC-IMC Lift	Z OMC-IMC Lift	Y OMC-IMC Lift	X OMC-IMC Lower	Z OMC-IMC Lower	Y OMC-IMC Lower
Differences in Spine-to-Load Distance (m)						
Mean	0.08	0.12	0.03	0.08	0.13	0.04
SD	0.05	0.07	0.03	0.05	0.07	0.04
Max	0.27	0.34	0.17	0.28	0.35	0.17
Min	0.00	0.00	0.00	0.00	0.00	0.00
25th percentile	0.04	0.07	0.01	0.04	0.07	0.01
50th percentile	0.07	0.11	0.03	0.07	0.12	0.03
75th percentile	0.11	0.16	0.04	0.11	0.17	0.05
90th percentile	0.15	0.21	0.07	0.15	0.22	0.08

**Table 3:** ANOVA results testing the effects of the load (A), angle (B), height (C), and interactions for the compressive and shear forces at L5-S1 during the lifting tasks. Significant effects (*p* < 0.05) are indicated in ***bold*** and ***italics***. Large effect sizes (ω^2^ > 0.14) are indicated in **bold**.

Lifting	Peak OMC		Peak IMC		RMSE between Systems		Peak Difference	
Factors	*p* Value	Effect Size ω^2^	*p* Value	Effect Size ω^2^	*p* Value	Effect Size ω^2^	*p* Value	Effect Size ω^2^
Force PD								
Groups	***<0.001***	**0.544**	***<0.001***	**0.551**	***0.049***	0.138	***0.005***	**0.262**
(A) Load	***<0.001***	**0.938**	***<0.001***	**0.873**	***<0.001***	**0.614**	***<0.001***	**0.767**
(B) Angle	0.117	0.007	0.280	0.002	0.216	0.005	0.121	0.021
(C) Height	0.499	<0.001	0.994	<0.001	***<0.001***	**0.234**	0.383	0.000
A × B	0.570	<0.001	0.062	0.022	0.146	0.014	0.120	0.032
A × C	0.974	<0.001	0.122	0.014	0.258	0.007	0.496	<0.001
B × C	0.404	<0.001	0.802	<0.001	0.144	0.014	0.57	<0.001
A × B × C	0.819	<0.001	0.420	0.001	0.639	<0.001	0.874	<0.001
Force AP								
Groups	***<0.001***	**0.518**	***<0.001***	**0.477**	***0.017***	**0.197**	***<0.001***	**0.397**
(A) Load	***<0.001***	**0.916**	***<0.001***	**0.868**	***<0.001***	**0.576**	***<0.001***	**0.576**
(B) Angle	0.399	<0.001	0.814	<0.001	0.155	0.010	0.079	0.041
(C) Height	0.282	0.002	0.994	<0.001	***<0.001***	0.259	0.121	0.029
A × B	0.864	<0.001	0.764	<0.001	0.574	<0.001	0.984	<0.001
A × C	0.827	<0.001	***0.047***	0.035	0.22	0.011	0.172	0.033
B × C	0.099	0.015	0.385	0.001	0.277	0.007	0.362	0.006
A × B × C	0.636	<0.001	0.796	<0.001	0.528	<0.001	0.252	0.032

**Table 4:** ANOVA results testing the effects of load (A), angle (B), height (C), and interactions for the compressive and shear forces at L5-S1 during the lowering tasks. Significant effects (*p* < 0.05) are indicated in ***bold*** and ***italics***. Large effect sizes (ω^2^ > 0.14) are indicated in **bold**.

Lowering	Peak OMC		Peak IMC		RMSE between Systems		Peak Difference	
Factors	*p* Value	Effect Size ω^2^	*p* Value	Effect Size ω^2^	*p* Value	Effect Size ω^2^	*p* Value	Effect Size ω^2^
Force PD								
Groups	***<0.001***	**0.475**	***<0.001***	**0.427**	***0.011***	**0.217**	***0.02***	**0.187**
(A) Load	***<0.001***	**0.826**	***<0.001***	**0.934**	***<0.001***	**0.630**	***<0.001***	**0.836**
(B) Angle	0.269	0.004	0.586	<0.001	0.454	<0.001	0.922	<0.001
(C) Height	0.848	<0.001	0.688	<0.001	***<0.001***	0.125	0.926	<0.001
A × B	0.274	0.008	0.077	0.012	0.051	0.035	0.236	0.019
A × C	0.541	<0.001	0.37	0.001	0.249	0.009	0.936	<0.001
B × C	0.695	<0.001	0.349	0.001	0.078	0.028	0.86	<0.001
A × B × C	0.183	0.024	0.721	<0.001	0.180	0.023	0.848	<0.001
Force AP								
Groups	***<0.001***	**0.457**	***<0.001***	**0.428**	***0.002***	**0.296**	***<0.001***	**0.386**
(A) Load	***<0.001***	**0.928**	***<0.001***	**0.875**	***<0.001***	**0.628**	***<0.001***	**0.698**
(B) Angle	0.655	<0.001	0.518	<0.001	0.604	<0.001	0.758	<0.001
(C) Height	0.574	<0.001	0.861	<0.001	***0.001***	**0.104**	0.913	<0.001
A × B	0.250	0.005	0.356	0.003	0.377	0.002	0.757	<0.001
A × C	0.364	0.001	0.576	<0.001	0.121	0.025	0.72	<0.001
B × C	0.316	0.003	0.407	<0.001	0.078	0.033	0.766	<0.001
A × B × C	0.913	<0.001	0.669	<0.001	0.143	0.034	0.712	<0.001

**Table 5:** ANOVA results testing the effects of load (A), angle (B), height (C), and interactions for joint angles during the lifting tasks. Significant effects (*p* < 0.05) are indicated in ***bold*** and ***italics***. Large effect sizes (ω^2^ > 0.14) are indicated in **bold**.

Lifting	Peak OMC		Peak IMC		RMSE between Systems		Peak Difference	
Factors	*p* Value	Effect Size ω^2^	*p* Value	Effect Size ω^2^	*p* Value	Effect Size ω^2^	*p* Value	Effect Size ω^2^
Flexion or Extension								
Groups	***<0.001***	**0.392**	***<0.001***	**0.369**	0.546	<0.001	0.783	<0.001
(A) Load	0.063	0.091	***0.028***	0.127	0.675	<0.001	0.886	<0.001
(B) Angle	***0.003***	**0.261**	***0.014***	**0.163**	0.578	<0.001	0.803	<0.001
(C) Height	0.435	<0.001	0.051	0.096	0.994	<0.001	0.449	<0.001
A × B	0.385	0.006	0.880	<0.001	0.279	0.054	0.222	0.096
A × C	0.676	<0.001	0.288	0.026	0.346	0.025	0.477	<0.001
B × C	0.250	0.037	0.557	<0.001	0.455	<0.001	0.811	<0.001
A × B × C	0.378	0.020	0.211	0.075	0.564	<0.001	0.661	<0.001
Lateral Bending								
Groups	0.14	0.074	0.674	<0.001	0.776	<0.001	0.251	0.035
(A) Load	0.093	0.039	0.733	<0.001	0.339	0.013	0.108	0.080
(B) Angle	***<0.001***	**0.626**	***<0.001***	**0.731**	0.715	<0.001	0.804	<0.001
(C) Height	0.549	<0.001	0.979	<0.001	0.493	<0.001	0.574	<0.001
A × B	0.415	<0.001	0.455	<0.001	0.578	<0.001	0.132	0.104
A × C	0.142	0.042	0.680	<0.001	0.933	<0.001	0.184	0.075
B × C	0.788	<0.001	0.373	0.004	0.601	<0.001	0.206	0.064
A × B × C	0.547	<0.001	0.265	0.027	0.755	<0.001	0.632	<0.001
Rotation								
Groups	0.672	<0.001	***<0.001***	**0.440**	0.240	0.038	***<0.001***	**0.478**
(A) Load	0.420	<0.001	0.558	<0.001	0.124	0.097	0.23	0.027
(B) Angle	***<0.001***	**0.7756**	***<0.001***	**0.802**	0.475	<0.001	0.071	0.096
(C) Height	***0.008***	0.0816	0.785	<0.001	0.600	<0.001	***0.018***	**0.180**
A × B	0.970	<0.001	0.409	0.001	0.784	<0.001	0.469	<0.001
A × C	0.909	<0.001	0.961	<0.001	0.471	<0.001	0.847	<0.001
B × C	0.650	<0.001	0.633	<0.001	0.500	<0.001	0.871	<0.001
A × B × C	0.824	<0.001	0.311	0.016	0.464	<0.001	0.141	0.134

**Table 6:** ANOVA results testing the effects of load (A), angle (B), height (C), and interactions for joint angles during the lowering tasks. Significant effects (*p* < 0.05) are indicated in ***bold*** and ***italics***. Large effect sizes (ω^2^ > 0.14) are indicated in **bold**.

Lowering	Peak OMC		Peak IMC		RMSE between Systems		Peak Difference	
Factors	*p* Value	Effect Size ω^2^	*p* Value	Effect Size ω^2^	*p* Value	Effect Size ω^2^	*p* Value	Effect Size ω^2^
Flexion or Extension								
Groups	***<0.001***	**0.361**	***<0.001***	**0.379**	0.494	<0.001	0.755	<0.001
(A) Load	0.111	0.093	0.076	0.094	0.542	<0.001	0.851	<0.001
(B) Angle	0.156	0.066	0.118	0.067	0.899	<0.001	0.412	<0.001
(C) Height	0.614	<0.001	***0.017***	**0.187**	0.694	<0.001	***0.002***	**0.369**
A × B	0.760	<0.001	0.753	<0.001	0.412	0.002	0.156	0.091
A × C	0.379	0.011	0.225	0.052	0.686	<0.001	0.867	<0.001
B × C	0.573	<0.001	0.504	<0.001	0.502	<0.001	0.422	<0.001
A × B × C	0.394	0.023	0.677	−0.062	0.715	<0.001	0.894	<0.001
Lateral Bending								
Groups	0.343	0.012	0.992	<0.001	0.728	<0.001	0.257	0.033
(A) Load	***0.020***	0.066	0.971	<0.001	0.438	<0.001	0.139	0.106
(B) Angle	***<0.001***	**0.654**	***<0.001***	**0.709**	0.641	<0.001	0.769	<0.001
(C) Height	0.517	<0.001	0.620	<0.001	0.461	<0.001	0.702	<0.001
A × B	0.382	0.003	0.776	<0.001	0.592	<0.001	0.422	<0.001
A × C	0.166	0.028	0.370	0.005	0.843	<0.001	0.774	<0.001
B × C	0.391	0.002	0.363	0.005	0.674	<0.001	0.367	0.020
A × B × C	0.634	<0.001	0.355	0.014	0.677	<0.001	0.897	<0.001
Rotation								
Groups	0.202	0.0497	***<0.001***	**0.373**	0.238	0.039	***<0.001***	**0.460**
(A) Load	0.158	0.0246	0.505	<0.001	0.128	0.086	***0.049***	0.087
(B) Angle	***<0.001***	**0.7390**	***<0.001***	**0.876**	0.130	0.085	***<0.001***	**0.463**
(C) Height	0.167	0.0231	0.929	<0.001	0.589	<0.001	0.501	<0.001
A × B	0.939	<0.001	0.343	0.004	0.921	<0.001	0.700	<0.001
A × C	0.942	<0.001	0.932	<0.001	0.637	<0.001	0.776	<0.001
B × C	0.633	<0.001	0.651	<0.001	0.537	<0.001	0.621	<0.001
A × B × C	0.775	<0.001	0.728	<0.001	0.336	<0.001	0.291	0.040

## Data Availability

Data are contained within the article and Supplementary Materials.

## Supplementary Materials

The following supporting information can be downloaded at https://www.youtube.com/watch?v=kKelr2xoFj0. Video S1: IMC vs. OMC study (trial example).

## Appendix A

**Table A1:** Summary of measures of the force estimates for both motion capture systems.

Summary Measure	Peak OMC Lift	Peak IMC Lift	Peak OMC Lower	Peak IMC Lower	RMSE Lift	RMSE Lower
Force PD (N)						
Mean	2798.52	1823.36	2644.29	1741.59	544.66	556.86
SD	666.71	433.61	614.82	391.68	156.83	161.03
Max	4864.02	3010.83	4262.28	2773.24	871.70	920.28
Min	1623.87	1063.77	1354.51	917.27	224.15	161.26
25th percentile	2279.24	1516.67	2214.72	1448.62	420.45	436.55
50th percentile	2708.36	1759.61	2628.07	1722.28	524.98	546.58
75th percentile	3195.15	2096.70	2963.76	1969.03	648.16	684.75
90th percentile	3651.78	2435.74	3524.45	2232.75	773.54	756.67
Force AP (N)						
Mean	944.99	653.22	898.91	624.99	147.83	154.68
SD	233.66	152.72	216.34	136.69	51.06	51.28
Max	1684.01	1103.53	1524.13	989.27	284.40	293.85
Min	532.51	393.01	485.71	354.05	47.06	39.49
25th percentile	770.15	549.01	742.61	532.08	109.62	121.24
50th percentile	920.16	627.84	887.49	600.62	144.97	149.71
75th percentile	1116.86	750.22	1032.33	715.50	178.16	187.52
90th percentile	1266.48	866.29	1214.76	807.75	223.52	225.44

**Table A2:** Summary of measures of the joint angles estimates for both motion capture systems.

Summary Measure	Peak OMC Lift	Peak IMC Lift	Peak OMC Lower	Peak IMC Lower	RMSE Lift	RMSE Lower
Joint Angles (Degrees)						
Flexion or Extension Angle						
Mean	62.62	44.64	63.68	46.52	10.92	11.19
SD	10.64	10.34	11.28	10.62	4.79	4.90
Max	81.91	63.47	86.57	65.02	28.18	28.55
Min	39.77	16.27	36.93	15.53	3.73	2.45
25th percentile	54.40	37.83	57.38	39.22	7.07	7.53
50th percentile	64.29	46.02	64.86	47.55	10.21	10.69
75th percentile	71.76	52.30	72.32	54.09	13.14	12.99
90th percentile	74.77	56.94	75.27	60.14	16.86	18.14
Lateral Bending Angle						
Mean	9.88	8.90	9.95	8.45	4.19	4.16
SD	5.11	4.09	4.76	3.86	2.12	2.16
Max	27.83	20.81	25.61	18.27	11.42	11.33
Min	3.08	2.61	3.03	2.57	1.04	0.93
25th percentile	6.56	5.66	6.62	5.59	2.75	2.61
50th percentile	8.78	8.24	9.08	8.22	3.70	3.74
75th percentile	10.96	11.18	11.63	10.41	5.01	5.17
90th percentile	16.94	15.12	16.33	13.93	7.21	7.24
Rotation Angle						
Mean	9.11	14.62	8.76	14.95	5.93	6.07
SD	4.41	6.51	4.23	7.21	3.19	2.97
Max	22.24	36.44	23.04	35.78	16.31	13.86
Min	2.23	4.91	2.56	3.77	0.95	1.02
25th percentile	6.11	9.80	5.46	9.21	3.61	3.93
50th percentile	7.74	14.01	7.80	13.45	5.05	5.65
75th percentile	11.51	18.80	11.41	19.16	7.54	7.77
90th percentile	14.58	22.09	14.58	23.88	9.67	10.22
